# Research Progress on Ammonia Sensors Based on Ti_3_C_2_T_x_ MXene at Room Temperature: A Review

**DOI:** 10.3390/s24144465

**Published:** 2024-07-10

**Authors:** Kaixin Cheng, Xu Tian, Shaorui Yuan, Qiuyue Feng, Yude Wang

**Affiliations:** 1 School of Materials and Energy, Yunnan University, Kunming 650091, China; ckx0316@126.com (K.C.); xutian@mail.ynu.edu.cn (X.T.); 18787877622@163.com (S.Y.); fengqiuyue@stu.ynu.edu.cn (Q.F.); 2 Yunnan Key Laboratory of Carbon Neutrality and Green Low-Carbon Technologies, Yunnan University, Kunming 650091, China

**Keywords:** Ti_3_C_2_T_x_ MXene, gas sensors, ammonia, room-temperature, sensitivity mechanism

## Abstract

Ammonia (NH_3_) potentially harms human health, the ecosystem, industrial and agricultural production, and other fields. Therefore, the detection of NH_3_ has broad prospects and important significance. Ti_3_C_2_T_x_ is a common MXene material that is great for detecting NH_3_ at room temperature because it has a two-dimensional layered structure, a large specific surface area, is easy to functionalize on the surface, is sensitive to gases at room temperature, and is very selective for NH_3_. This review provides a detailed description of the preparation process as well as recent advances in the development of gas-sensing materials based on Ti_3_C_2_T_x_ MXene for room-temperature NH_3_ detection. It also analyzes the advantages and disadvantages of various preparation and synthesis methods for Ti_3_C_2_T_x_ MXene’s performance. Since the gas-sensitive performance of pure Ti_3_C_2_T_x_ MXene regarding NH_3_ can be further improved, this review discusses additional composite materials, including metal oxides, conductive polymers, and two-dimensional materials that can be used to improve the sensitivity of pure Ti_3_C_2_T_x_ MXene to NH_3_. Furthermore, the present state of research on the NH_3_ sensitivity mechanism of Ti_3_C_2_T_x_ MXene-based sensors is summarized in this study. Finally, this paper analyzes the challenges and future prospects of Ti_3_C_2_T_x_ MXene-based gas-sensitive materials for room-temperature NH_3_ detection.

## 1. Introduction

NH_3_ is a colorless, alkaline gas with a pungent odor that is now widely used in industry, agriculture, and other fields such as refrigerant and nitrogen fertilizer production. Excessive NH_3_ emissions are closely related to the biological environment and the health of humans, such as the formation of acid rain and some human diseases [1,2]. According to the Occupational Safety and Health Administration (OSHA), when the human body is exposed to 35 ppm of NH_3_ for more than 15 min, this will endanger human health, and when the concentration of NH_3_ reaches 500 ppm, people will suffer from acute toxicity, eye pain, shortness of breath, and other symptoms, as well as the risk of asphyxiation [3,4]. In addition, NH_3_ can be a marker gas for human diseases such as kidney, liver, or H. pylori infections [5], and NH_3_ can be used for the early prevention of diseases and monitoring of the disease process via changes in the concentration of exhaled NH_3_ in the human body [6].

A gas sensor is a sensitive device capable of converting the gas concentration that needs to be measured into an electrical signal. By analyzing the electrical signal, the sensor can obtain information such as the gas concentration present in the environment [7,8,9]. In recent decades, traditional commercialized NH_3_ gas-sensitive materials have focused on metal oxide semiconductors (MOS) such as SnO_2_, TiO_2_, WO_3_, ZnO, and other materials. Scientists have conducted many studies on them because they have high gas-response values [10]. However, most of the MOS-based NH_3_ sensors operate at temperatures above 100 °C, and the higher operating temperature and their consumption of large amounts of energy limit their applications at room temperature and the flexible wearable applications of NH_3_ [11]. Therefore, the development of a sensing material that can realize the real-time and efficient detection of NH_3_, which exhibits strong sensitivity and a rapid response and can work at room temperature, is urgently needed for production safety and human health [12].

Two-dimensional (2D) transition metal carbons and nitrides (MXenes) are emerging 2D materials with a graphene-like layered structure with the general formula M_n+1_X_n_T_x_, where the “M” stands for the early transition metals, such as Ti, V, Ta element, etc.; the “X” is the C or N element; the “n” is 1, 2, or 3; and the “T” is the surface functional group, such as hydroxyl (-OH), oxygen (-O), fluorine (-F), chlorine (-Cl), and so on [13]. MXenes were first discovered by Prof. Yury Gogotsi in 2011 [14]. Their excellent electrical conductivity, large specific surface area, and rich surface functional groups that provide more active sites for gas adsorption and reaction have attracted widespread attention and led to their use in energy storage, photovoltaic devices, supercapacitors, and gas sensors [15,16,17]. Ti_3_C_2_T_x_ is one of the first and most extensively studied MXene materials [18]. It has been shown both theoretically and experimentally that Ti_3_C_2_T_x_ MXene has excellent NH_3_ sensitivity at room temperature [19]. Density functional theory (DFT) calculations have shown that the -F, -O, and -OH groups on the surface of Ti_3_C_2_T_x_ MXene materials have high adsorption energies with NH_3_. It was also shown that Ti_3_C_2_T_x_ MXene materials are effective at sensitizing gases to NH_3_ [20,21].

However, it is the high adsorption energy between Ti_3_C_2_T_x_ MXene and NH_3_ that also leads to the resistance drift and long recovery time from the NH_3_ environment to the air. NH_3_ sensors based on pure Ti_3_C_2_T_x_ MXene often exhibit poor selectivity, slow response and recovery times, and low response values [22,23]. So, in order to obtain an excellent NH_3_-sensitive performance at room temperature, scientists have paid a lot of attention and conducted a lot of research focused on the choice of synthesis method, composite modification, and systematic sensitization mechanism of Ti_3_C_2_T_x_ MXene-based ammonia sensors.

In this context, this paper begins with Ti_3_C_2_T_x_ MXene synthesis methods, provides a classified overview of the most recent research progress in Ti_3_C_2_T_x_ MXene synthesis, which is primarily classified as HF etching, in situ HF etching and alkali solution etching, and summarizes the benefits and drawbacks of the various etching techniques. In addition, several types of composites, including metal–oxide semiconductors, conductive polymers, and 2D material composites, are briefly discussed in order to enhance the NH_3_-sensitive behavior of NH_3_ sensors based on pure Ti_3_C_2_T_x_ MXene. These Ti_3_C_2_T_x_ MXene composites are further analyzed to prove that the primary mechanisms of the enhanced NH_3_-sensing performance of these sensors are the synergistic effects between the composites, such as the formation of a heterojunction of the composites, adsorption energy and charge transfer, chemical sensitization and electron sensitization, and the increase in specific surface area and adsorption sites due to their unique morphology [24,25].

Finally, this paper reviews the research progress regarding the sensitization mechanism of NH_3_ sensors based on Ti_3_C_2_T_x_ MXene, then summarizes and discusses the two current sensitization mechanisms that can explain the p-type response of MXene to either electron acceptor or electron donor gases: (1) MXene is metallic in nature, and the number of carriers decreases and the electrical resistance increases after the gas adsorption. When they exhibit semiconductor properties, the charge transfer mechanism follows the Wockenstein model [26,27,28]. (2) When the MXenes are exposed to the gas, the interlayer expansion hinders electron transfer and the resistance increases [16]. Finally, the paper summarizes the key challenges and potential paths forward for NH_3_ sensors based on Ti_3_C_2_T_x_ MXene. We expect that this review will provide novel ideas for the development of high-performance, room-temperature Ti_3_C_2_T_x_ MXene NH_3_ sensors.

## 2. Synthesis of Ti_3_C_2_T_x_ MXene

The majority of Ti_3_C_2_T_x_ MXene materials are produced by selectively etching the precursor MAX phase (a hexagonal layered structure with the common structural formula M_n+1_A_n_X_n_, where the “n” = 1, 2, or 3; examples of such materials are Ti_2_AlC, Ti_3_AlC_2_, Ti_2_SiC, Ti_4_AlN_3_, etc.; the “A” is a group IIIA or IVA element [29,30]). The A atomic layer is more easily etched away during the reaction with the etchant, leaving a 2D layered structure with alternating M and X layers because the M-A bonds in the MAX phase are stronger than the M-X bonds in terms of bond strength.

**Figure 1:**
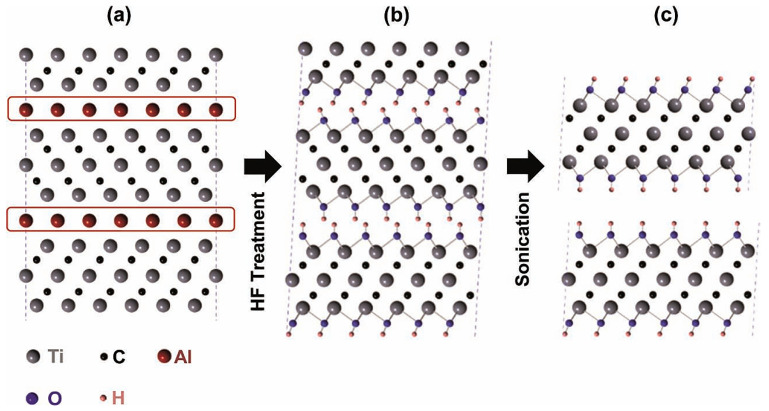
Ti_3_AlC_2_ is etched as Ti_3_C_2_T_x_. (**a**) Structural schematic diagram of Ti_3_AlC_2_, (**b**) schematic of the process by which -OH replaces Al atoms after HF treatment, and (**c**) hydrogen bond breaking and nanolayer separation after sonication treatment [14].

The functional groups -OH, -O, and -F in the etching system are easily attached to the surface of this alternating layer structure, eventually forming a 2D layered structure, Ti_3_C_2_T_x_ MXene [31,32]. Ti_3_AlC_2_ is most used in the MAX phase, as shown in Figure 1, which presents a schematic diagram of the etching process of Ti_3_AlC_2_ by HF, where the Ti_3_AlC_2_ structure consists of a single Ti_3_C_2_ layer separated by Al atoms. The Al atom layers between the Ti_3_AlC_2_ layers are eliminated by the HF treatment, and the subsequent loss of metallic bonds causes the individual Ti_3_Al_2_C layers to separate, producing Ti_3_C_2_T_x_. The reaction process is shown in Equations (1)–(3):

(1) $${Ti}_{3}AlC_{2}+3HF=AlF_{3}+3/2H_{2}+{Ti}_{3}C_{2}$$

(2) $${Ti}_{3}C_{2}+2H_{2}O={Ti}_{3}C_{2}(OH{)}_{2}+H_{2}$$

(3) $${Ti}_{3}C_{2}+2HF={Ti}_{3}C_{2}F_{2}+H_{2}$$

The substitution of the -F functional group represented by Equation (3) is similar to that of -OH; therefore, its reaction mechanism can be considered to regard -OH in Figure 1 as -F.

The different etching methods and the choice of etchant can change the types and ratios of the functional groups at the end of the Ti_3_C_2_T_x_ MXene material, thus affecting the adsorption behaviors of Ti_3_C_2_T_x_ MXene and NH_3_ and influencing the NH_3_-sensitive performance of the sensors. In the current study, most Ti_3_C_2_T_x_-based NH_3_-sensing materials are synthesized using HF etching and in situ HF etching. In addition, there are a small number of research papers that mention the alkaline solution etching method. Therefore, this chapter will briefly describe these three etching methods and their effects on ammonia gas-sensing.

### 2.1. HF Etching

**Figure 2:**
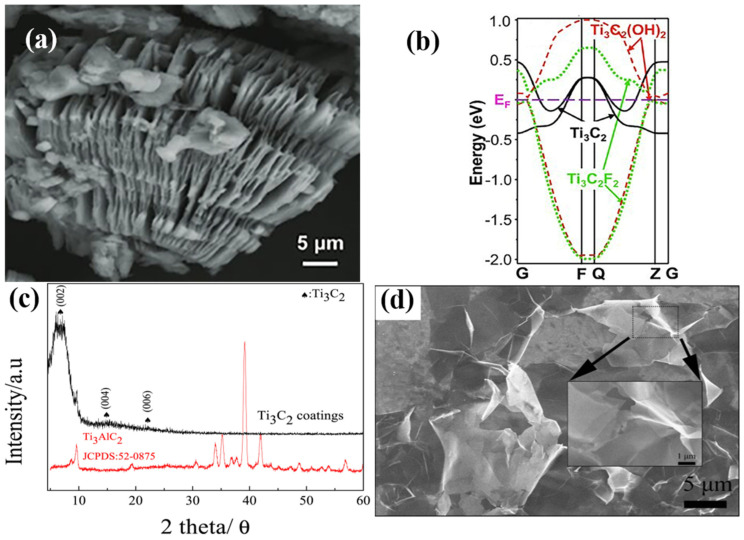
(**a**) SEM image of the Ti_3_C_2_T_x_ MXene after HF treatment; (**b**) by comparing the single-layer band structures of Ti_3_C_2_(OH)_2_, Ti_3_C_2_F_2_, and Ti_3_C_2_, it can be seen that Ti_3_C_2_T_x_ exhibits a change from metal to semiconductor due to changes in surface functional groups [14]; (**c**) XRD pattern of Ti_3_AlC_2_ and as-prepared Ti_3_C_2_T_x_; (**d**) SEM images of Ti_3_C_2_ nanoflakes after exfoliation by TMAOH [33].

HF etching is a useful approach for preparing Ti_3_C_2_T_x_ MXene, which can be created by etching the MAX phase. In 2011, Naguib et al. [14] prepared the first 2D Ti_3_C_2_ phase MXene by etching Ti_3_AlC_2_ powder with high concentrations of HF. Figure 2a shows an HFtreated SEM image of Ti_3_AlC_2_, revealing that the Ti_3_C_2_T_x_ basal plane is accordion-like with a fan-like dispersion. The energy band structure of the synthesized MXene (Ti_3_C_2_) terminated with the −OH and −F end groups was calculated, and the results displayed that the mechanism of the electrical conductivity of the Ti_3_AlC_2_ material changed from metallic to semiconducting after etching due to a change in its surface chemistry (Figure 2b). Due to the simplicity of the HF etching method, it remains one of the most commonly used etchants for synthesizing Ti_3_C_2_T_x_ MXene.

The specific surface area of Ti_3_C_2_T_x_ MXene has a great influence on its gas-sensitive properties. The gas-sensitive properties can be improved by increasing the specific surface area of Ti_3_C_2_T_x_ Mxene through intercalation and layering. For the further intercalation and delamination of Ti_3_C_2_T_x_ obtained by HF etching, Lian et al. [33] reported a method involving 2D Ti_3_C_2_T_x_ nanosheets synthesized by intercalation and a delamination reaction in tetramethylammonium hydroxide (TMAOH) solution after dilute HF etching. They added a quantitative amount of Ti_3_AlC_2_ powder to a 5% concentrated HF solution, which was magnetically stirred at room temperature for 24 h. Then, the suspension was washed with deionized water until the pH of the supernatant was 5–6 and centrifuged. After that, the precipitate was put in a 25% solution of tetramethyl hydroxide (TMAOH) and magnetically agitated for another 24 h to allow for more intercalation and delamination. Afterward, the precipitate was centrifuged to obtain the two-dimensional, less-layered Ti_3_C_2_T_x_ nanosheets. Figure 2c displays the XRD spectra of Ti_3_AlC_2_ and Ti_3_C_2_. After HF etching, the (104) diffraction peak of Ti_3_AlC_2_ at 2θ ≈ 39.1° almost disappeared, which indicated that the Ti_3_C_2_ was completely stripped [34,35]. The SEM image of the Ti_3_C_2_ nanoflakes intercalated with TMAOH is shown in Figure 2d. A high level of delamination is indicated by the two-dimensional structure, semi-transparent, and wrinkled textures of the Ti_3_C_2_ nanoflakes. In future research, we can increase the specific surface area of Ti_3_C_2_T_x_ MXene-sensitive materials through the intercalation and delamination of Ti_3_C_2_T_x_ to increase the adsorption sites on the surface of the materials to promote their adsorption and gas reaction with ammonia molecules, ultimately aiming to achieve the purpose of improving the sensitivity of Ti_3_C_2_T_x_ MXene.

### 2.2. In-Situ HF Etching

Although the HF etching process is easy to operate, it has obvious disadvantages, such as a longer etching time and a more dangerous operation [36]. Furthermore, the Ti_3_C_2_T_x_ MXene obtained by this process has more shortcomings: HF is dangerous to humans and the environment, and fluorine-containing functional groups are inert, degrading their properties. In 2014, Ghidiu et al. [37] presented a method to synthesize Ti_3_C_2_T_x_ MXene by etching the Ti_3_AlC_2_ phase in situ with LiF and HCl solution. Specifically, LiF was added to a 6 M HCl solution and stirred to dissolve before slowly adding Ti_3_AlC_2_ powder and maintaining this at 40 °C for 45 h. After that, the mixture was washed until the pH of the supernatant was 6. The obtained deposit formed a clay-like paste, which was rolled between the roller mill’s permeable membranes to produce pliable, freestanding thin films within a few minutes. When diluted, it can be applied as an ink to deposit or print MXene on various substrates.

During the reaction process, Li^+^ can spontaneously insert into the interlayer of Ti_3_C_2_T_x_ MXene, and due to the hydrophilicity of Ti_3_C_2_T_x_ MXene, water molecules can easily enter the interlayer, leading to a much larger lattice parameter for Ti_3_C_2_T_x_ MXenes obtained by LiF/HCl etching than for Ti_3_C_2_T_x_ MXenes obtained by HF etching. This process produces large-component monolayer Ti_3_C_2_T_x_ flakes with a high yield, large lateral size, and excellent quality. More importantly, using LiF and HCl to etch MAX avoids the use of concentrated HF, which is very corrosive and poisonous, while also reducing the nanoscale defects generated by direct etching with HF. Furthermore, the simplicity of this method contrasts with previous films produced through laborious insertion, layering, and filtration techniques [38]. It is noteworthy that MXenes can still be obtained when H_2_SO_4_ is used instead of HCl. However, fine-tuning the etching reagent might affect the surface chemistry or the intercalation ions, which needs more exploration. Currently, in situ etching by LiF and HCl solutions is widely used in the synthesis of Ti_3_C_2_T_x_ MXene materials.

Han et al. [39] compared the ammonia sensitivity of Ti_3_C_2_T_x_ MXene materials synthesized by HF etching and LiF/HCl etching. They found that the Ti_3_C_2_T_x_ materials prepared by the LiF/HCl etchant had better gas sensitivity than that of HF etchant. They could detect a wide range of NH_3_ at room temperature with higher sensitivity and stability. The higher sensing performance of the Ti_3_C_2_T_x_ MXene materials obtained by the LiF/HCl etching method was analyzed to be due to the high ratio of -O and -OH functional groups on the surface of Ti_3_C_2_T_x_ materials prepared by this method.

### 2.3. Alkali Solution Etching

**Figure 3:**
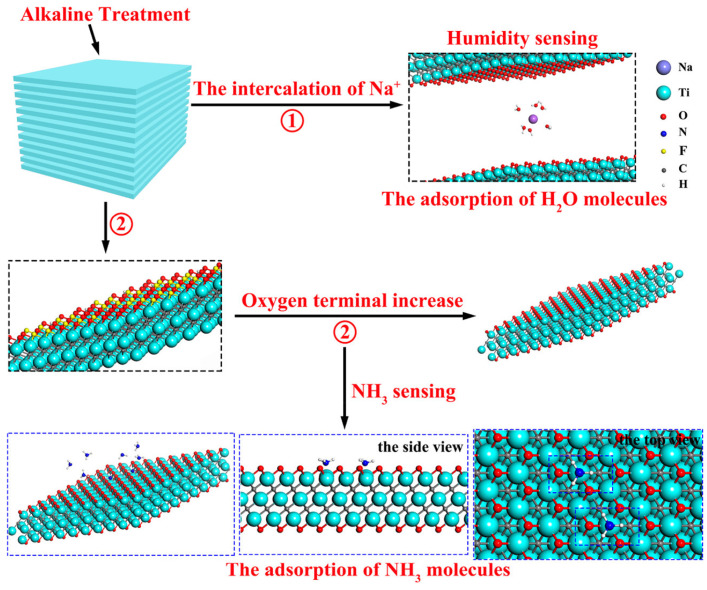
Adsorption process of H_2_O and NH_3_ molecules on the surface of alkalized Ti_3_C_2_T_x_ [40].

Yang et al. [40] improved the humidity and NH_3_-sensing properties of organ-like Ti_3_C_2_T_x_ MXene synthesized from HF solution via alkalization. Organ-like Ti_3_C_2_T_x_ MXene is a class of Ti_3_C_2_T_x_ MXene materials that can provide a fast pathway for charge and ion transfer, thereby preventing the reduction in specific surface area due to the restacking of layers. Firstly, Ti_3_C_2_T_x_ MXene powder was prepared through HF acid etching using Ti_3_AlC_2_ and a 45 wt% HF solution. Subsequently, the obtained Ti_3_C_2_T_x_ MXene powder was put into a 5 M NaOH solution and subjected to continuous magnetic stirring at room temperature for 2 h, and the alkalized Ti_3_C_2_T_x_ powder was finally obtained. The alkali treatment embeds Na^+^ in the interlayer of Ti_3_C_2_T_x_, which plays a crucial role in regulating water for humidity sensing. During stage 1 of Figure 3, several water molecules are coupled with a single Na^+^ ion, forming a stable [Na(H_2_O)_m_]^+^ cluster structure. Meanwhile, in stage 2 of Figure 3, the alkaline treatment enhances the N-Ti bonding sites due to the increased -O terminus, resulting in higher NH_3_ adsorption. The alkalized Ti_3_C_2_T_x_ devices have an improved sensing performance for humidity and NH_3_ compared to non-alkalized Ti_3_C_2_T_x_, due to the embedding of Na^+^ and the higher oxygen-to-fluorine atomic number ratio ([O]/[F]), and have the opposite response signal.

Although there is no report on the synthesis of Ti_3_C_2_T_x_ MXene ammonia-sensitive materials using only alkali etching, this method allows for the preparation of Ti_3_C_2_T_x_ MXene with a large number of oxygen-containing functional groups, is simple to perform, and does not contain inert groups -F. Therefore, it is expected to be applicable to the synthesis of Ti_3_C_2_T_x_ MXene-based ammonia-sensitive materials in the future and to obtain sensitive materials with a strong ammonia-adsorption capacity.

Ti_3_C_2_T_x_ MXene synthesized by conventional fluoride-containing methods usually uses fluoride ion-containing solutions as an etchant, which is harmful to the environment. In addition, highly reactive fluoride ions can penetrate into the human body and cause fatal damage to body tissues [41,42]. It has been demonstrated that fluoride-based etching procedures generate a large number of -F-terminal functional groups [43,44], which substantially limit charge transfer and diminish chemically active sites, hence compromising the electrochemical characteristics of Ti_3_C_2_T_x_ MXene [45,46,47]. Therefore, modulating the terminal functional groups of Ti_3_C_2_T_x_ MXene can affect its electrochemical properties and NH_3_ sensitivity [48]. Furthermore, the popular HF and LiF/HCl etching methods are typically time-consuming [49]. Therefore, it is important to develop environmentally friendly and F-free synthesis routes for NH_3_ sensor-based Ti_3_C_2_T_x_ MXene in the future.

## 3. Ti_3_C_2_T_x_ MXene-Based Nanocomposites Material Gas Sensors for NH_3_

Ti_3_C_2_T_x_ Mxenes are a typical new 2D material with high selectivity for ammonia at room temperature. Dillon, Lipatov et al. [35,50] measured the high electrical conductivity of Ti_3_C_2_T_x_ MXene to be 6500 S cm^−1^ and 4600 ± 1100 S cm^−1^. Halim et al. [51] have also reported that Ti_3_C_2_ films exhibit metal conductivity at a temperature of about 100 K. Ti_3_C_2_T_x_ MXenes is considered to have good application prospects for ammonia detection at room temperature due to its excellent electrical conductivity, unique structure, and the large number of adsorption sites provided by functional groups such as -O and -OH on the surface [52,53,54]. Due to the low response values, serious base-resistance drift, and poor stability of pure Ti_3_C_2_T_x_ MXenes when utilized as ammonia-sensing materials, it is essential to combine Ti_3_C_2_T_x_ MXene with other sensitive materials to enhance their sensing capabilities [25,55,56]. In this chapter, a brief overview will be provided of the most-researched methods of compositing several sensitive materials with Ti_3_C_2_T_x_ MXenes to improve their sensing performance. These methods include combining them with MOS materials, conductive polymer materials, and certain 2D materials.

### 3.1. Metal Oxide Modification

MOS materials such as TiO_2_, SnO_2_, In_2_O_3_, WO_3_, and Ti_3_C_2_T_x_ MXene usually use synergistic effects such as the Fermi energy level effect (Fermi level bending, carrier separation, depletion layer regulation, and increase in interfacial barrier energy) between the composites, the formation of heterojunctions, and the specific morphology, etc., to enhance the gas-sensitive performance of the sensors [57,58,59]. In particular, for TiO_2_/Ti_3_C_2_T_x_ MXene nanocomposites, in addition to the introduction of TiO_2_ material in the matrix, due to the instability of Ti_3_C_2_T_x_ MXene, TiO_2_can be synthesized through partial oxidization to Ti_3_C_2_T_x_ MXene, leading to in situ derivatization using techniques such as high-temperature sintering.

When a semiconductor material is irradiated by UV light whose optical energy is larger than its band gap, photocarrier pairs are generated at the interface of the sensitive material [60,61], and the additionally generated photogenerated electrons and holes can promote the adsorption and desorption processes and redox reactions of gases, thus improving the response value and shortening the response recovery time of the sensor [62,63]. Therefore, using the UV irradiation of gas sensors to obtain highly NH_3_-sensitive Ti_3_C_2_T_x_ MXene-based sensors operating at room temperature is a feasible strategy.

**Figure 4:**
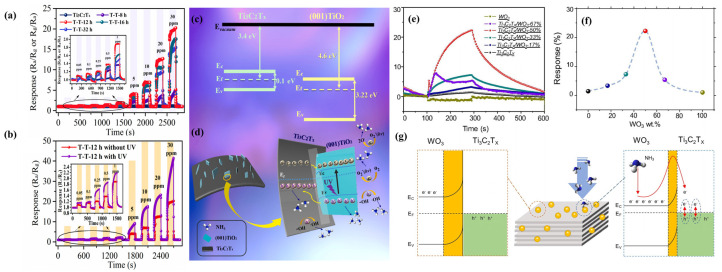
(**a**) Dynamic response curves of TT8 h-, TT12 h-, TT16 h- and TT32 h-based sensors; (**b**) dynamic response curves with UV illumination on T-T-12 h and without UV illumination on TT12 h; (**c**) schematic illustration of band diagrams of Ti_3_C_2_T_x_ and (001) TiO_2_; (**d**) schematic illustration of ammonia gas-sensing mechanism under UV irradiation [64]. (**e**) The transient response curves of the composite sensors with different WO_3_ contents to 1 ppm NH_3_ at room temperature; (**f**) the dependence of the composite sensor response on the WO_3_ content; (**g**) schematic illustration of the gas-sensing mechanism and the energy band structure diagram of Ti_3_C_2_T_x_/WO_3_ before and after exposed NH_3_ [70].

To overcome the limitations of Ti_3_C_2_T_x_ MXene as a room-temperature ammonia gas sensor, such as limited its sensitivity and selectivity, Zhang et al. [64] proposed growing TiO_2_ on Ti_3_C_2_T_x_ MXene in situ. They also utilized UV light (the light energy of the used 365 nm UV light source is 3.4 eV, which is larger than the band gap 1.6 eV of (001)TiO_2_/Ti_3_C_2_T_x_) to boost the performance of the (001) TiO_2_/MXene heterostructure ammonia gas sensor. The Ti_3_C_2_T_x_ powder was prepared by removing the Al layer from MAX (Ti_3_AlC_2_) with a 50% HF solution. NaBF_4_ (0.1 mol/L, 8 mL) was introduced as a control agent for the crystal surface in the HCl solution of Ti_3_C_2_T_x_. The mixture was then hydrothermally treated at 160 °C for 8, 12, 16, and 32 h. The hydrothermal reaction promoted the formation of (001) planes during the crystal growth process, converting Ti in Ti_3_C_2_ into hydrated Ti_3_^+^ ions and binding F ions to the (001) planes with the aid of NaBF_4_ as an inducer. As shown in Figure 4a, the T-T-12 h TiO_2_/MXene sensor that undergoes a hydrothermal reaction for 12 h is more sensitive to ammonia, with a lower detection limit than that of the pure Ti_3_C_2_T_x_-based sensor, and also exhibits good durability in terms of its response/recovery time, repeatability, and selectivity. Analyzing the energy band diagram schematic of the T-T-12 h TiO_2_/MXene-based sensor demonstrated in Figure 4c, it can be concluded that the enhanced gas-sensitive performance of NH_3_ is obtained because Ti_3_C_2_T_x_ greatly facilitates the separation of electron–hole pairs by storing holes through the Schottky junctions formed at the interfaces with TiO_2_, which enhances the ammonia-sensing performance. In Figure 4d, we can see that adding UV light to the highly active (001) crystalline TiO_2_ makes the charge separation of the T-T-12 h TiO_2_/MXene sensor when exposed to UV light even better, which leads to a better gas-sensing performance. Figure 4b also shows a two-fold increase in sensitivity compared to when UV is absent. The TiO_2_/MXene sensor type T-T-12 h exhibited 34 times more sensitivity to 30 ppm of ammonia compared to pristine Ti_3_C_2_T_x_. Density functional theory reveals that the (001) side of the TiO_2_ and Ti_3_C_2_T_x_ composite has the greatest attraction in terms of ammonia adsorption [64].

To further improve the sensing performance of pure Ti_3_C_2_T_x_ MXene for NH_3_, Kan et al. [65] combined surface-functionalized In_2_O_3_ nanotubes with Ti_3_C_2_T_x_ nanosheets. The composite was further loaded onto thermoplastic polyurethane (TPU) foam, a dual-functional sensing platform constructed based on Ti_3_C_2_T_x_/In_2_O_3_ nanocompositesand modified TPU foam sensors. The produced nanocomposites exhibited an improved ammonia-sensing performance and pressure-sensitive properties, utilizing the strong synergistic effect of the sensing of In_2_O_3_ nanotubes and high conductivity of Ti_3_C_2_T_x_ nanoflakes, as well as the foam substrate’s pressure-sensitive and gas-permeable capabilities. Ti_3_C_2_T_x_ nanoflakes are negatively charged on the surface due to the terminal functional groups such as -OH and -F. At this time, the modification of In_2_O_3_ with the cationic surfactant (3-aminopropyl) triethoxysilane (APTES) can make the surface of In_2_O_3_ positively charged, and the two can be compounded to obtain Ti_3_C_2_T_x_/In_2_O_3_ composites via simple ultrasonication with electrostatic adsorption, which was shown to have higher NH_3_ response values than pure Ti_3_C_2_T_x_ nanoflakes. In_2_O_3_ is an n-type sensing material, which has a bandgap in the range of 3.55–3.75 eV [66] and a work function of about 4.28 eV [67]; Ti_3_C_2_T_x_ MXene behaves as a p-type sensing material, which has a bandgap of 0.19 eV [68] and a work function of about 4.5 eV [69]. Two main factors are used to investigate the enhanced sensing ability: First, n-type In_2_O_3_ has a high Fermi energy level, and electrons will be transferred from the In_2_O_3_ nanotubes to the Ti_3_C_2_T_x_ nanoflakes until the Fermi energy levels of the two reach equilibrium, thus forming a space charge layer at the Ti_3_C_2_T_x_ and In_2_O_3_ interface. At the same time, the energy bands at the interface are bent on both sides, generating a potential barrier that increases the base value resistance of the sensor, thus increasing the gas response. Second, the In_2_O_3_ nanotubes work as isolators to inhibit the re-stacking of Ti_3_C_2_T_x_ nanoflakes, thereby increasing the layer space and effectively facilitating the diffusion and permeation of gases in the sensing layer. In addition, the sensor achieves the flexible and interference-free detection of complex exhaled environments at room temperature, with a memory function for detecting NH_3_ gases down to 1 ppm, realizing the dual-mode detection of NH_3_ gases.

To address the limited sensitivity of Ti_3_C_2_T_x_ MXene gas sensors, Guo et al. [70] reported a Ti_3_C_2_T_x_/WO_3_ composite resistive sensor that had excellent NH_3_ sensitivity at room temperature. Ti_3_C_2_T_x_/WO_3_ composites with WO_3_ nanoparticles anchored on Ti_3_C_2_T_x_ nanosheets were prepared using an ultrasonic technique. As demonstrated in Figure 4e,f, the detection sensitivity of the Ti_3_C_2_T_x_/WO_3_-50% sensor was up to 22.3%, which was 15.4 times higher than that of the pure Ti_3_C_2_T_x_ sensor, at 1 ppm NH_3_ at room temperature. The improved NH_3_-sensing performance is mainly attributed to the increase in the aspect ratio of the composite, the increase in the active sites provided by WO_3_, and the more effective charge transfer bestowed by the formed heterojunction (Figure 4g). It was demonstrated that the Ti_3_C_2_T_x_/WO_3_-50% sensor remained effective over a wide range of relative humidity (RH) conditions (3.5%~72.9% RH), and low concentrations of NH_3_ were still detected even at high humidity. As RH increases, the sensor response to NH_3_ sensing gradually decreases. Humidity compensation methods, which are often used in practical applications, can help to solve the humidity effect’s limitations.

Table 1 outlines the gas-sensitive properties of different MOS/Ti_3_C_2_T_x_ MXene composites towards NH_3_ at room temperature in recent years. The acronym “LOD” in the table refers to “the limit of detection”.

### 3.2. Conductive Polymer Addition

Conducting polymers, such as polyaniline (PANI), polypyrrole (PPy), polydioxothiophene (PEDOT), and their derivatives, possess the advantages of high flexibility, the ability to work at room temperature, and a high response to ammonia. As a result, they are receiving increasing attention for their use in NH_3_ sensing at room temperature [77,78]. To address the issues of Ti_3_C_2_T_x_ MXene resistance drift, the long recovery time, the low response value, and susceptibility to mechanical deformation, it has been proposed to hybridize Ti_3_C_2_T_x_ MXene with conductive polymers. This approach aims to enhance the sensitivity and wearable performance at room temperature of Ti_3_C_2_T_x_ MXene through the heterojunctions formed between the two materials and the high flexibility of the conducting polymers [79,80].

**Figure 5:**
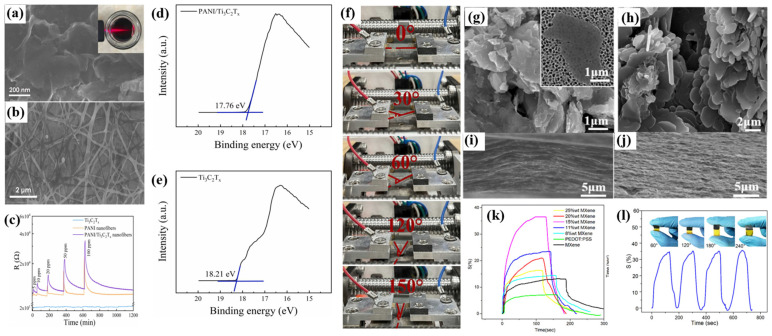
(**a**) SEM image of Ti_3_C_2_T_x_ MXene; (**b**) SEM image of PANI/Ti_3_C_2_T_x_; (**c**) PANI/Ti_3_C_2_T_x_ composite nanofiber sensor for the detection of NH_3_ at room temperature; (**d**) UPS spectra of pure Ti_3_C_2_T_x_ sensor and (**e**) PANI/Ti_3_C_2_T_x_ composite sensors; (**f**) the PANI/Ti_3_C_2_T_x_-based flexible sensor bent at different angles [82]. FESEM images of (**g**) Ti_3_C_2_T_x_ MXene (inset is Ti_3_C_2_T_x_ MXene on an AAO membrane) and (**h**) PEDOT:PSS/Ti_3_C_2_T_x_ MXene composites. The cross-sectional FESEM images of (**i**) Ti_3_C_2_T_x_ MXene films and (**j**) PEDOT:PSS/Ti_3_C_2_T_x_ MXene films, (**k**) effect of the Ti_3_C_2_T_x_ MXene content in PEDOT-PSS on the sensor response toward 100 ppm NH_3_ at room temperature (27 °C), and (**l**) gas response of the PEDOT:PSS/Ti_3_C_2_T_x_ MXene composite-based sensor against 100 ppm NH_3_ bent at different angles [94].

Polyaniline (PANI) has been widely used in room-temperature ammonia sensors due to its strong electrical conductivity, superior selectivity to ammonia, and room-temperature workability [81,82]. Yang et al. [82] used electrostatic spinning to combine polyaniline with Ti_3_C_2_T_x_ nanosheets to construct polyaniline/Ti_3_C_2_T_x_ composite nanofibers with excellent ammonia responsiveness for room-temperature flexible ammonia sensors. The SEM image in Figure 5a demonstrates that the synthesized Ti_3_C_2_T_x_ exhibits a distinct layer structure, and the Tyndall phenomenon can be seen in the inset. Figure 5b is an SEM image of PANI/Ti_3_C_2_T_x_, which reveals a 3D network fiber structure with random fiber orientation. Figure 5c shows that the PANI/Ti_3_C_2_T_x_ flexible sensor has a greater NH_3_-sensing response at 25 °C (2.3 times the response value at 20 ppm compared to pure PANI), with good selectivity, repeatability, and long-term stability. Figure 5f demonstrates that by adjusting the bending angle (0°–150°) and the number of bending times (up to 3200), a high sensing performance at 20 ppm NH_3_ and enduring flexible bending stability can be attained. PANI is a conductive p-type semiconductor with a band gap of about 2.47 eV [83]; the resistance of the pure Ti_3_C_2_T_x_ sensor, exhibiting a metallic nature, was tested to be about 24 Ω [79], and the Ti_3_C_2_T_x_ band gap was reviewed in the literature to be about 0.19 eV [68]. In the UPS results of the composites in Figure 5d,e, the figures of merit for Ti_3_C_2_T_x_ and PANI/Ti_3_C_2_T_x_ composite nanofibers are 2.99 eV and 3.44 eV, respectively (UV is He I, 21.22 eV). Therefore, the formation of the Schottky junction at the interface between Ti_3_C_2_T_x_ and PANI enhances the resistance modulation of the flexible sensor. The gas sensitivity of the PANI/Ti_3_C_2_T_x_ flexible sensor was shown to be enhanced because the composite of the PANI and Ti_3_C_2_T_x_ nanosheets constitutes a Schottky junction, the degree of PANI protonation is increased, and the two are hybridized to form a special three-dimensional reticulated fibrous structure, which has great potential for the construction of flexible ammonia gas sensors.

In addition, electrostatic spinning has been widely used in conductive polymer sensors as an easy, efficient, low-cost nanofiber preparation technique. Compared with the traditional synthesis method, the electrostatic spinning method can greatly increase nanomaterials’ specific surface area volume ratio [84]. By integrating polymers with nanofiber structures into resistive gas sensors, porous materials with a high surface area and large porosity can be prepared to enhance the adsorption of ammonia molecules by the sensors and improve the sensitivity of the sensors, as well as to further optimize the properties of the polymer-sensitive materials, such as their hydrophobicity, conductivity, and flexibility [85,86]. In addition, these sensors also showed fast response and recovery times. Zhang synthesized polyaniline and polymethyl methacrylate (PMMA) nanocomposite fibers using electrostatic spinning [87]. This sensitive material was shown to have a fast response and high sensitivity to NH_3_, with a sensitivity of 4.5% to 5 ppm NH_3_ and a response/recovery time of about 5 s/12 s. On this basis, through the composite of different conductive polymer materials and Ti_3_C_2_T_x_ MXene, as well as the optimization of the electrostatic spinning process conditions, the development of NH_3_-sensitive materials with a high sensing performance is expected. This is essential for further optimizing the ammonia sensor performance of the conducting polymer/Ti_3_C_2_T_x_ MXene composites.

Poly (3,4-ethylenedioxythiophene):polystyrene sulfonate (PEDOT:PSS) has attracted a lot of attention in the field of gas sensing because of its good electrical conductivity, low bandgap, environmental friendliness, capacity to detect dangerous chemicals at low operating temperatures [88,89,90,91], and environmental stability [92,93]. Jin et al. [94] created PEDOT:PSS/Ti_3_C_2_T_x_ MXene composites by combining 3,4-ethylenedioxythiophene (PEDOT) and poly (4-styrenesulfonate) (PSS) on a Ti_3_C_2_T_x_ MXene material on a polyimide (PI) substrate through in situ polymerization. Figure 5g,h show the SEM images of Ti_3_C_2_T_x_ MXene and PEDOT:PSS/Ti_3_C_2_T_x_ MXene composites, respectively, where the Ti_3_C_2_T_x_ exhibits a monolayered or few-layered lamellar structure after the introduction of the PEDOT:PSS, where the surface of Ti_3_C_2_T_x_ MXene is covered by micro- and submicrometer PEDOT:PSS plates. Comparing Figure 5i,j reveals that the introduction of PEDOT:PSS increases the gap between the layers of the composite material. The gas sensitivity of NH_3_ achieved the highest response value (Figure 5k) when Ti_3_C_2_T_x_ MXene contained 15 wt% and the fastest recovery/response with 36.6% response to 100 ppm NH_3_ at room temperature, a 116 s response time, and a 40 s recovery time. Furthermore, Figure 5l shows a bending test of the sensor at a maximum angle of 240°, and the gas response of the device does not change with the change in the bending angle, indicating that it has good mechanical stability.

The mechanism for analyzing the gas-sensitive performance enhancement of the PEDOT:PSS/Ti_3_C_2_T_x_ MXene composite mainly involves the redox reaction between the composite and the analyte, the charge transfer between the composite and the analyte, and synergistic effect of the increase in the specific surface area of the composite materials. Compared with pure PEDOT:PSS and Ti_3_C_2_T_x_ MXene-based sensors, the composite sensors have a high gas response, fast response/recovery, a low detection limit, good reproducibility, high selectivity, and excellent mechanical stability. A number of studies have demonstrated that the NH_3_ sensors prepared from Ti_3_C_2_T_x_ MXene and conductive polymer hybrid materials have a low detection limit, flexibility, and an excellent sensing performance [95].

Table 2 outlines the gas-sensing properties of different conductive polymer /Ti_3_C_2_T_x_ MXene composites towards NH_3_ at room temperature that were determined in recent years.

### 3.3. Other 2D Material Hybrids

In addition to MXene, graphene, as well as various emerging 2D materials such as transition metal disulfide compounds (TMDs), reduced graphene oxide (rGO), and black phosphorus (BP), have been rapidly developed for NH_3_-sensing due to their graphene-like layered structure, large specific surface area, semiconducting properties, and room-temperature operation. It has been demonstrated that the heterojunction constructed by compositing Ti_3_C_2_T_x_ MXenes with these 2D materials can obtain complementary electrical and adsorption properties, which enhances their sensing performance [95].

**Figure 6:**
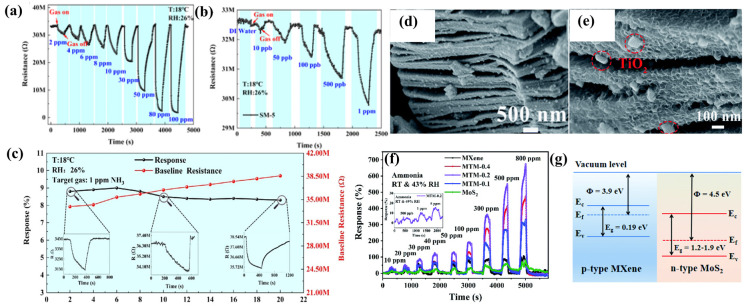
The resistance variation curves of the SM-5 sensor to (**a**) ppm-level NH_3_ concentration from 2 to 100 ppm and (**b**) ppb-level NH_3_ concentration from 10 ppb to 1 ppm at 18 °C; (**c**) the long-term stability of the SM-5 sensor to 1 ppm NH_3_ for 20 days [56]. (**d**,**e**) Ti_3_C_2_T_x_ MXene@TiO_2_/MoS_2_; (**f**) dynamic sensing performance of the sensor-based Ti_3_C_2_T_x_ MXene to NH_3_ at a room temperature of 27 °C and an RH of 43%; (**g**) energy band diagrams of Ti_3_C_2_T_x_ MXene@TiO_2_/MoS_2_ sensors [30].

It has been demonstrated that when MXenes are combined with TMDs, such as molybdenum disulfide (MoS_2_), tin disulfide (SnS_2_), tungsten disulfide (WS_2_), and other transition metal disulfide compounds, their charge transfer and adsorption abilities are enhanced, which leads to better sensing effects [100,101]. In addition, the composite of TMDs and MXene has almost no lattice mismatch [102], and is expected to replace the traditional metal oxide composite with MXene to construct high-performance ammonia gas-sensitive materials [103,104]. In order to overcome the difficulty of detecting ultra-low concentrations of ammonia at room temperature in chemical-based gas sensors, He et al. [56] developed an MXene/SnS_2_ heterojunction-type chemoresistive sensor SM-5 (the nominal weight ratio of MXene/SnS is 1:5), as shown in Figure 6a,b, which exhibits an excellent gas-sensitive performance for ammonia at sub-ppm at room temperature. The SM-5 sensor can detect NH_3_ concentrations of as low as 10 ppb at room temperature. In addition, Figure 6c shows the excellent long-term stability of the sensor, with a decrease in response value of about 3.4% in 20 days. Meanwhile, the SM-5 sensor showed good selectivity to a wide range of possible interfering gases, such as HCHO, C_2_H_5_OH, CH_3_OH, C_3_H_6_O, C_6_H_6_, and NO_2_. The sensing mechanism of the MXene/SnS_2_-based sensor is closely related to the formation of heterostructures. DFT calculations show that the higher sensitivity and selectivity may be due to the more effective charge transfer bestowed by the formed heterostructure, the better catalytical activity, and the stronger NH_3_ adsorption of the formed MXene/SnS_2_ composite material.

Our group [30] demonstrated a Ti_3_C_2_T_x_ MXene@TiO_2_/MoS_2_ nanocomposite gas sensor, which successfully realized the detection of 500 ppb NH_3_ at room temperature. MoS_2_ nanosheets were grown in situ on the etched Ti_3_C_2_T_x_ MXene material via the hydrothermal method, and then some of the Ti_3_C_2_T_x_ MXene was converted into rectangular TiO_2_ particles through hydrothermal reaction. The SEM images of Ti_3_C_2_T_x_ MXene@TiO_2_/MoS_2_ at different scales are shown in Figure 6e; it can be observed that rectangular TiO_2_ nanoparticles are attached between the layers of the material and on the surface, which increase the spacing of the layers of the composite material and can provide more reaction sites for the target gas. As shown in Figure 6f, this novel gas sensor (MTM-0.2) based on a layered structure exhibits the advantages of a fast response and high stability in detecting NH_3_. At 100 ppm ammonia, the composite gas sensor had 1.79 and 2.75 times higher response values than the pristine Ti_3_C_2_T_x_ MXene and MoS_2_. Furthermore, at room temperature, the Ti_3_C_2_T_x_ MXene@TiO_2_/MoS_2_ nanocomposite gas sensor exhibited high selectivity for triethylamine, trimethylamine, n-butanol, acetone, formaldehyde, and nitrogen dioxide. As shown in Figure 6g, MoS_2_ is an n-type sensing material, which has a bandgap in the range of 1.2–1.9 eV [105] and a work function of about 3.9 eV [106]. Ti_3_C_2_T_x_ MXene behaves as a p-type sensing material, which has a bandgap of 0.19 eV [68] and a work function of about 4.5 eV [69]. The heterojunction of the p-type Ti_3_C_2_T_x_ MXene and n-type MoS_2_ was the primary reason for the sensor’s improved gas-sensitive performance for NH_3_. Bader charge analysis can further obtain the adsorption between sensitive materials and gases by evaluating the amount of charge transfer between gas molecules and sensitive material models [107]. Bader analysis further confirmed that ammonia molecules increase charge transfer in the heterojunction, which enhances the interaction of Ti_3_C_2_T_x_ MXene@TiO_2_/MoS_2_ nanocomposites with ammonia.

**Figure 7:**
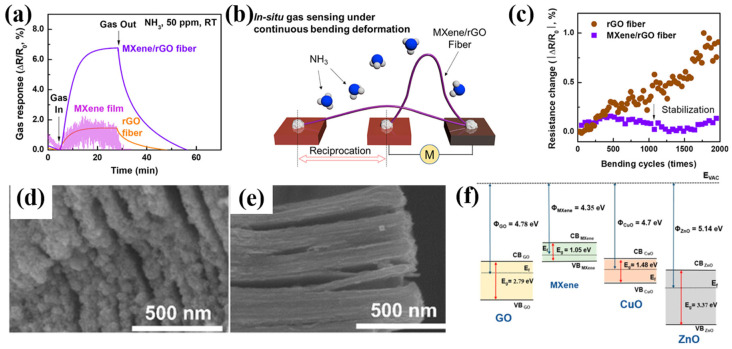
(**a**) Comparison of the gas response of MXene film, rGO fiber, and MXene/rGO hybrid fiber; (**b**) schematic illustration of the fiber bending test. The “M” stands for multimeter. (**c**) Cyclic bending fatigue versus resistance difference of the rGO fiber and MXene/rGO hybrid fiber [19]. (**d**) FE-SEM images of Ti_3_C_2_T_x_ MXene/GO/CuO/ZnO nanocomposite and (**e**) pristine Ti_3_C_2_T_x_ Mxene. (**f**) Schematic diagram of the energy band structure of the Ti_3_C_2_T_x_ MXene/GO/CuO/ZnO heterostructure [25].

Graphene fibers (GFs) have a high mechanical flexibility, electrical conductivity, and wear ability and have enormous potential for application in wearable electronic devices [108,109]. Lee et al. [19] reported a simple, scalable, and effective strategy for wet-spinning MXene/graphene-based hybrid fibers using a wet-spinning process to obtain metal-free binder Ti_3_C_2_T_x_ MXene/graphene hybrid fibers. These hybrid fibers have excellent mechanical and electrical properties, making them suitable for flexible, wearable gas sensors. The synergistic effect of the electronic properties and gas adsorption capacity of MXene/graphene resulted in a high NH_3_ gas sensitivity at room temperature. Figure 7a compares the response values of MXene, rGO fiber, and MXene/rGO fiber sensors, and shows that the Ti_3_C_2_T_x_ MXene/graphene hybrid fiber exhibited a significantly improved NH_3_-sensing response (Δ*R*/*R*_0_ = 6.77%). The hybrid fiber exhibits excellent mechanical flexibility. Figure 7b depicts a schematic diagram of the device used to detect the stability of the fiber optic sensor by bending it repeatedly and detecting the change in the resistance of the sensor. As shown in Figure 7c, the resistance fluctuation remains small, ±0.2%, even after more than 2000 bends. In addition, the highly flexible MXene/rGO hybrid fiber was woven/knitted into the lab coat using a simple conventional weaving procedure, demonstrating a dependable sensing capability. The synergistic effect of the optimized bandgap and the enhanced atomic oxygen content at the MXene end of the MXene/rGO hybrid fiber significantly improved the NH_3_-sensing response performance of the MXene/rGO hybrid fiber with low power consumption.

Yotsarayuth et al. [25] successfully synthesized Ti_3_C_2_T_x_ Mxene/GO/CuO/ZnO nanocomposites by mixing common NH_3_-sensing materials such as graphene oxide (GO), copper oxide (CuO), and zinc oxide (ZnO) into Ti_3_C_2_T_x_ Mxene via a simple and low-cost hydrothermal method. Figure 7d shows the SEM images of the composites, and the Ti_3_C_2_T_x_ Mxene/GO/CuO/ZnO nanocomposites exhibit a highly uniform two-dimensional stacked structure. A comparison with the SEM image of pure Ti_3_C_2_T_x_ MXene in Figure 7e reveals that the composites have a high surface roughness, which enhances the active sites for the adsorption of NH_3_ gas molecules. Figure 7f depicts Ti_3_C_2_T_x_ MXene, GO, CuO, and ZnO figures of work functions of 4.35, 4.78, 4.7, and 5.14 eV [19,110,111,112], respectively, with Ti_3_C_2_T_x_ MXene having the lowest figure of work function. As a result, electrons are transferred from Ti_3_C_2_T_x_ MXene to the other three materials to achieve Fermi energy level equilibrium, forming multiple p-n heterojunctions at the interfaces of the different materials. Ti_3_C_2_T_x_ MXene/GO/CuO/ZnO exhibits a 59.9% response to 100 ppm NH_3_ at room temperature, with a response time of 26 s and a recovery time of 25 s. The Ti_3_C_2_T_x_ MXene/GO/CuO/ZnO exhibits excellent selectivity, high responsiveness, good repeatability, strong stability, a quick response recovery time, and humidity independence. The Ti_3_C_2_T_x_ MXene/GO/CuO/ZnO gas sensor exhibits a remarkable NH_3_-sensing performance. This is because of the inherent features and properties of the nanocomposites, including their functional groups, their bonding, the strong intermolecular attraction between NH_3_ molecules, the nanocomposites, and the formation of a p-n heterojunction.

Table 3 outlines the gas-sensitive properties of different 2D materials/Ti_3_C_2_T_x_ MXene composites towards NH_3_ at room temperature in recent years.

## 4. Ti_3_C_2_T_x_ MXenes-Based Nanocomposite Material Mechanism for NH_3_

The unique properties of terminating functional groups on the surface of MXenes make them ideal for ammonia-sensitive materials. Bhardwaj et al. [115] found that the effective adsorption energy of Ti_2_CO_2_ with NH_3_ was −0.37 eV using DFT calculations, and the charge transfer between monolayer Ti_2_CO_2_, which has a similar structure to that of Ti_3_C_2_O_2_ and NH_3_, was 0.174 e. Similarly, Atkare et al. [95] discovered that MXenes, including monolayers of Ti_3_C_2_O_2_, Ti_2_C(OH)_2_, and others, exhibit a significant affinity to NH_3_, resulting in a charge transfer of 0.117 e.

At room temperature, the reaction mechanism of Ti_3_C_2_T_x_ MXene to NH_3_ molecules can consist of two parts: the reaction between NH_3_ molecules and oxygen molecules on the surface of the sensitive material and the reaction between NH_3_ molecules and specific functional groups at the end of Ti_3_C_2_T_x_ MXene [18,57]. First, the adsorption of O_2_ molecules on the surface of the sensitive material traps electrons in the form of oxygen ions, mainly in the form of O^2−^. When the sensor is exposed to a reducing gas such as NH_3_, the O^2−^ will react with the NH_3_ molecules to form NO and H_2_O while releasing electrons. The resistance decreases when these electrons return to the conduction band of Ti_3_C_2_T_x_ MXene and its composites. The reaction process is shown in Equations (4)–(6):

(4) $$O_{2}\;(\mathrm{gas})\to O_{2}\;(\mathrm{ads})$$

(5) $$O_{2}\left(ads\right)+e^{-}\to O_{2}^{-}$$

(6) $$4{NH}_{3}+O_{2}^{-}\to 4NO+6H_{2}O+5e^{-}$$

**Figure 8:**
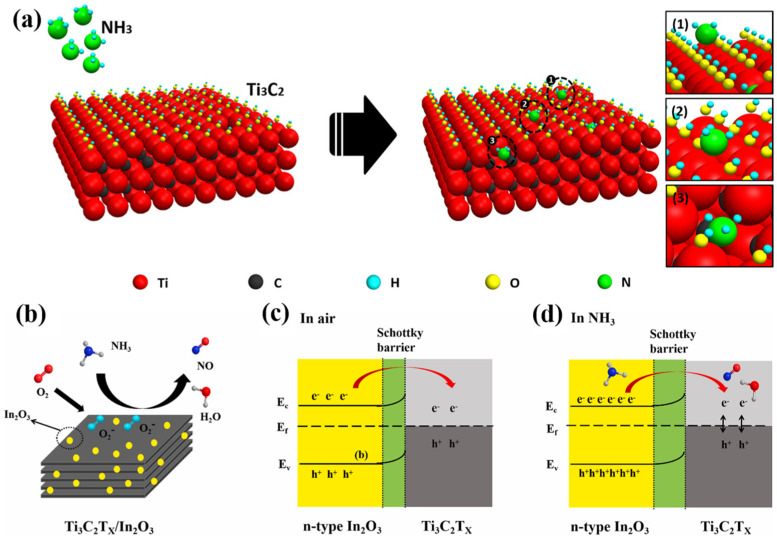
(**a**) Schematic diagram of the possible gas-sensing mechanisms of the Ti_3_C_2_T_x_ MXene for NH_3_ [18]. (**b**) The gas-sensing mechanism diagram of the Ti_3_C_2_T_x_/In_2_O_3_ composite materials. (**c**,**d**) Schematic diagram of the electron transfer at the interface of Ti_3_C_2_T_x_/In_2_O_3_ composite materials in the air and NH_3_ [57].

The gas-sensing mechanism of Ti_3_C_2_T_x_/In_2_O_3_ composites to NH_3_ shown in Figure 8b can be used as a reference. In addition to the electron transfer process between oxygen ions and NH_3_ molecules on the surface of Ti_3_C_2_T_x_ MXene-sensitive materials, Lee et al. [57] mentioned that the reaction of -O and -OH on the surface of Ti_3_C_2_T_x_ with NH_3_ resulted in hole–electron complexation and a subsequent increase in resistance, and the conjectured mechanism diagram is shown in Figure 8a. The reaction formulae are shown in Equations (7) and (8) as follows:

(7) $$2{NH}_{3}+3O^{-}\to N_{2}+3H_{2}O+3e^{-}$$

(8) $$NH_{3}+OH^{-}\to NH_{2}+H_{2}O+e^{-}$$

The mechanism for the enhanced gas sensitivity of Ti_3_C_2_T_x_ MXene composite materials to NH_3_ is generally achieved by the synergistic effect between the two materials, such as the formation of a heterojunction of the composites, adsorption energy and charge transfer, chemical sensitization and electron sensitization, unique morphology, etc. [11,61]. When heterojunctions are formed, the gap in the work function between Ti_3_C_2_T_x_ MXene and its composite material causes electrons to transfer directionally from the lower to the higher work function. This transfer occurs due to the equilibrium of the Fermi energy levels when the materials come into contact with each other. This results in the formation of an electron depletion layer and an electron accumulation layer on the low-work-function material. The electron accumulation layer promotes oxygen adsorption on the surface of the sensing material. In contrast, the electron depletion layer increases the potential energy barrier at the interface, while the height of the intergranular barrier hinders the transport of carriers, resulting in an increase in the initial resistance [116] and a higher gas response. As an illustration, Zhou et al. [57] synthesized Ti_3_C_2_T_x_/In_2_O_3_ nanocomposites. In_2_O_3_ is classified as an n-type semiconductor, while Ti_3_C_2_T_x_ is regarded as a metallic phase because of its excellent conductivity. When Ti_3_C_2_T_x_ comes into contact with In_2_O_3_, it exhibits a high work function. As a result, electrons migrate from In_2_O_3_ to Ti_3_C_2_T_x_, forming a Schottky barrier and a depletion layer at the interface of In_2_O_3_ and Ti_3_C_2_T_x_ (as shown in Figure 8c,d). As a result, the charge transfer is blocked, and the base-value resistance of Ti_3_C_2_T_x_/In_2_O_3_ becomes high in the air, resulting in a significant increase in the gas-sensitive response [22]. In addition, the improved gas-sensitive performance of the sensitive material is also related to its lattice mismatch, and the oxygen vacancies generated at the heterojunction due to the lattice mismatch will also provide additional active sites for the sensitive material [117].

The most significant characteristic of MXenes is their demonstration of similar metallic properties to their precursor MAX, with a fixed electron density near the Fermi energy level. In fact, the gas-sensing mechanism of MXene is notably more complex than the classical charge transfer model [95]. Lee et al. [18] were the first to report the gas-sensing mechanism of Ti_3_C_2_T_x_ MXene. They observed increased resistance in Ti_3_C_2_T_x_ sensors when exposed to four electron donor gases (ethanol, methanol, acetone, and ammonia). Conversely, the resistance of Ti_3_C_2_T_x_ films decreased without the presence of these gases. Therefore, they inferred that Ti_3_C_2_T_x_ films exhibit p-type sensing behavior. The analysis suggests that the p-type semiconductor nature of Ti_3_C_2_T_x_ MXene may be due to the many molecules introduced during the Al etching process acting as p-type dopants to Ti_3_C_2_T_x_, such as water and oxygen.

It is well known that gas sensors for semiconductor materials exhibit either a positive or negative resistance change depending on the gas type [118]. Specifically, an n-type semiconductor-sensitive material exposed to oxidizing gases decreases in resistance due to the loss of electrons and increases due to the gain in electrons when exposed to reducing gases, and the opposite is true for p-type semiconductor-sensitive materials. In contrast, Kim et al. [118] found that Ti_3_C_2_T_x_ exhibits a positive resistance change under oxidized or reduced gas conditions, indicating that the carrier transport of Ti_3_C_2_T_x_ will be blocked when it adsorbs gases. In other words, the sensing mechanism of Ti_3_C_2_T_x_ is different from that of semiconductor materials such as metal oxides. Therefore, the mechanism for the universal p-type response of Ti_3_C_2_T_x_ was proposed. Due to the metallic conductivity of Ti_3_C_2_T_x_ MXene [119], the gas adsorption reduces the number of carriers and therefore increases the channel resistance. While Koh et al. [120] investigated the effect of the interlayer swelling of Ti_3_C_2_T_x_ MXene films upon gas action on the gas-sensitive properties, the change in the interlayer space of Ti_3_C_2_T_x_ MXene upon the introduction of gas was studied by in situ XRD measurements, which found that the degree of swelling of Ti_3_C_2_T_x_ MXene films was consistent with the gas response. Therefore, another mechanism is proposed: gas-phase molecules are inserted into the MXene interlayer instead of surface adsorption, and the interlayer expansion induced to reduce the conductivity due to the metallic nature of Ti_3_C_2_T_x_ MXene is one of the reasons why MXene generally exhibits a p-type response to various gases.

Both mechanisms currently explain MXene’s p-type response to the electron acceptor gas and the electron donor gas. To date, the gas-sensing mechanism of Ti_3_C_2_T_x_ MXene and its composites has not been explained in a unified manner. The electron transfer model of the adsorbed gas molecules on the surface of Ti_3_C_2_T_x_ composites may be more complex than reported and thus needs to be further developed and refined through experimental validation.

## 5. Conclusions and Outlook

Ti_3_C_2_T_x_ MXene, as the earliest discovered MXene material, which has been extensively studied in the field of ammonia gas sensing, has been regarded as an excellent room-temperature NH_3_-sensing material due to its graphene-like two-dimensional lamellar structure, which confers a large specific surface area, good room-temperature sensitivity, and a high adsorption capacity between its surface-rich functional groups and NH_3_. This review describes the current research progress in modulating the ammonia gas-sensing properties of Ti_3_C_2_T_x_ MXene by means of its preparation method and composite modification. Ti_3_C_2_T_x_ MXene with differences in its morphology, surface functional groups, electrochemical properties, nano-defects, and stability can be obtained through improvements in the preparation method to achieve the desired performance, which in turn affects the NH_3_-sensing behavior of the sensors. Ti_3_C_2_T_x_ MXene-based composites incorporating additional composites can demonstrate enhanced response values, lower detection limits, quicker response recovery, and an improved stability compared to pure Ti_3_C_2_T_x_ MXene.

Although significant progress has been made in the design and modification of Ti_3_C_2_T_x_ MXene materials for ammonia gas sensors in recent years, there are still many challenges and much room for further development in optimizing and improving the sensing performance. Since the gas-sensing mechanism of MXenes is much more complex than the traditional semiconductor classical charge-transfer model, further calculations and investigations regarding the charge transfer and adsorption–desorption are needed in terms of the NH_3_-sensing principle of Ti_3_C_2_T_x_ MXene. Meanwhile, a large number of studies have demonstrated that the surface-functional groups of Ti_3_C_2_T_x_ MXene have a significant effect on its electrochemical properties and stability. Therefore, synthesizing Ti_3_C_2_T_x_ MXene materials with controllable surface-functional groups is significant for its application in room-temperature ammonia gas sensors. Furthermore, Ti_3_C_2_T_x_ MXene’s composite structure can be further optimized to improve its NH_3_-sensing performance. In the next step, we could design Ti_3_C_2_T_x_ MXene sensors with excellent long-term stability and a response value that is less impacted by humidity to widen their applications. In the future, we should broaden our research on Ti_3_C_2_T_x_ MXene in these fields to develop higher-performance Ti_3_C_2_T_x_ MXene-based room-temperature ammonia sensors.

## Figures and Tables

**Table 1:** The gas-sensitive properties of various MOS/Ti_3_C_2_T_x_ MXene nanocomposites to NH_3_ at room temperature.

Materials	Concentration (ppm)	Response Value (R_a_/R_g_, R_g_/R_a_) or Response Rate [(ΔR/R_g_) × 100%]	LoD	Response/Recovery Time (s)	Ref./Year
NiO/Ti_3_C_2_T_x_	50	6.13	10 ppm	60/19	[71]/2023
Ti_3_C_2_T_x_/In_2_O_3_	30	63.8%	2 ppm	42/209	[57]/2022
TiO_2_/Ti_3_C_2_T_x_	10	3.1%	0.5 ppm	33/277	[22]/2019
Ti_3_C_2_T_x_/WO_3_	1	22.3%	1 ppm	119/228	[70]/2021
Ti_3_C_2_T_x_/CuO	5	46.7%	5 ppm	12/25	[72]/2023
ZnO/Ti_3_C_2_T_x_	20	39.16%	89.41 ppb	92/104	[58]/2023
In_2_O_3_/Ti_3_C_2_T_x_	5	60.6%	5 ppm	3/2	[73]/2022
Ti_3_C_2_T_x_/ZnO	50	196%	1 ppm	119/307	[74]/2023
Ti_3_C_2_T_x_/TiO_2_	30	40.6%	5 ppm	10/5	[64]/2022
Ti_3_C_2_T_x_/SnO_2_	50	40%	0.5 ppm	36/44	[68]/2021
Ti_3_C_2_T_x_/SnO	10	67%	1 ppm	61/119	[75]/2022
Ti_3_C_2_T_x_/TiO_2_/CuO	100	56.9%	10 ppm	75/80	[54]/2023
α-Fe_2_O_3_/Ti_3_C_2_T_x_	5	18.3%	5 ppm	2.5/2	[76]/2022

**Table 2:** The gas-sensitive properties of various conductive polymer/Ti_3_C_2_T_x_ MXene nanocomposites to NH_3_ at room temperature.

Materials	Concentration (ppm)	Response Value (R_a_/R_g_, R_g_/R_a_) or Response Rate [(ΔR/R_g_) × 100%]	LoD	Response/Recovery Time (s)	Ref./Year
PANI/Ti_3_C_2_T_x_	10	1.6	25 ppb	—	[79]/2020
PANI:PSS/Ti_3_C_2_T_x_	1	57%	20 ppb	276/388	[80]/2023
PANI/Ti_3_C_2_T_x_	20	55.9%	5 ppm	—	[82]/2023
PEDOT:PSS/N-Ti_3_C_2_T_x_	10	13%	10 ppm	—	[96]/2022
PANI/Ti_3_C_2_T_x_/TiO_2_	10	2.3	20 ppb	266/342	[24]/2023
Polyacrylamide/Ti_3_C_2_T_x_	200	4.7%	—	12.7/14.6	[97]/2020
PPy/MXene	100	31.9%	5 ppm	38/383	[98]/2022
PEDOT:PSS/Ti_3_C_2_T_x_	100	36.6%	10 ppm	116/40	[94]/2020
Ti_3_C_2_T_x_/PDDS	0.5	2.2%	500 ppb	—	[99]/2022

**Table 3:** The gas-sensitive properties of various 2D material /Ti_3_C_2_T_x_ MXene nanocomposites to NH_3_ at room temperature.

Materials	Concentration (ppm)	Response Value (R_a_/R_g_, R_g_/R_a_) or Response Rate [(ΔR/R_g_) × 100%]	LoD	Response/Recovery Time (s)	Ref./Year
Ti_3_C_2_T_x_@TiO_2_/MoS_2_	100	163.3%	500 ppb	117/88	[30]/2022
Ti_3_C_2_T_x_/SnS_2_	10	42.9%	10 ppb	161/80	[56]/2023
Ti_3_C_2_T_x_/rGO	50	6.77%	10 ppm	—	[19]/2020
Ti_3_C_2_T_x/_GO/CuO/ZnO	100	59.9%	4.1 ppm	26/25	[25]/2023
Ti_3_C_2_T_x_/MoS_2_	100	81.7%	200 ppb	3/—	[110]/2022
Ti_3_C_2_T_x_/TiO_2_/graphene	50	36.8%	22.23 ppb	19/29	[113]/2024
SnS/Ti_3_C_2_T_x_	5	1.031	250 ppb	7/—	[114]/2022
